# Microstructure, Tensile, and Creep Behaviors of Ti-22Al-25Nb (at.%) Orthorhombic Alloy with Equiaxed Microstructure

**DOI:** 10.3390/ma11071244

**Published:** 2018-07-20

**Authors:** Wei Wang, Weidong Zeng, Yaling Sun, Haixiong Zhou, Xiaobo Liang

**Affiliations:** 1 State Key Laboratory of Solidification Processing, Northwestern Polytechnical University, Xi’an 710072, China; 2 School of Metallurgy Engineering, Xi’an University of Architecture and Technology, Xi’an 710055, China; sunyaling@live.xauat.edu.cn (Y.S.); haixiong@live.xauat.edu.cn (H.Z.); 3 Beijing Iron & Steel Research Institute, Beijing 100081, China; liangxb_68@sina.com.cn

**Keywords:** Ti_2_AlNb-based alloy, equiaxed microstructure, tensile deformation, creep behavior

## Abstract

This article investigates the tensile and creep behaviors of the Ti-22Al-25Nb (at.%) alloy with equiaxed microstructure. The experimental results show that the equiaxed microstructures are formed by isothermal forging in the α_2_ + B2 + O phase region, and then heat treating in α_2_ + B2 + O and B2 + O phase regions. The equiaxed particles are determined by isothermal forging and solution heat treating, and the acicular O phase is obtained by adjusting the aging temperature. The strengths of the alloy are sensitive to the thickness of the secondary acicular O phase. Increase in aging temperature improves strength and reduces the ductility. Deformation of the alloy mainly depends on the volume fraction and deformability of the B2 phase. During the high-temperature tensile deformation, the flow stress decreases with the increasing deformation temperature and increases with the increasing strain rate. The microstructure obtained by higher aging temperature (HT-840) has better creep resistance, due to the coarsening of the secondary acicular O phase.

## 1. Introduction

Ti-Al-Nb alloys, based on the ordered orthorhombic (O) phase, have been considered potential materials for advanced aerospace applications. This includes use as aircraft engine parts, due to their excellent comprehensive performances, including high specific strength, low density, high fracture toughness, and outstanding creep and oxidation resistances [1,2]. Compared with conventional titanium alloys and TiAl-based intermetallics, Ti_2_AlNb based alloys have superior balanced mechanical properties (elevated-temperature strength, creep resistance and toughness) [3]. The phase structure of the O phase alloys is different from that of commercial titanium alloys. It includes several different phase types: orthorhombic phase (O) (Cmcm system based on Ti_2_AlNb), hexagonal close-packed (hcp) α_2_ phase (DO_19_ structure base on Ti_3_Al) and bcc phase β (disordered body-centered cubic structure) or B2 phase (ordered body-centered cubic structure) [1,2,3]. In recent years, the alloy with nominal chemical composition Ti-22Al-25Nb (at.%), which primarily consisted of an O + B2 two-phase microstructure, was reported to have optimum comprehensive mechanical properties [4,5,6,7,8].

The mechanical properties of Ti_2_AlNb-based alloys are important criteria for material service in the aerospace industry [9]. While the mechanical properties are determined by the microstructures, optimization and control of the microstructure are important for understanding the relationships among the processing technology, microstructure, and mechanical properties. Previous investigations revealed that different thermal processes and subsequent heat treatments presented different morphological changes including equiaxed, duplex, near lath, and fully lath microstructures in Ti-22Al-25Nb alloy [7,8,9,10,11,12]. The lath microstructures, including fully lamellar or nearly lamellar, are mainly contained in the O and B2 phases. The creep resistances and fracture toughness of lath microstructures are better than that of the duplex microstructure. Compared with lath, duplex have better ductility and strength, but poorer fracture toughness and creep resistance [13,14,15]. The equiaxed microstructure is a typical characteristic in Ti_2_AlNb-based alloys. However, studies on the tensile and creep behaviors of equiaxed microstructures in the Ti-22Al-25Nb alloys are very rare, especially high temperature (exceeding 650 °C) tensile behaviors. The deformation mechanisms of the equiaxed microstructure are still in the early stage of research, and much work remains.

In this study, we investigate the tensile and creep behaviors of the equiaxed microstructure in Ti-22Al-25Nb alloy. The deformation mechanisms of the equiaxed microstructure at room temperature will be discussed. The effects of the deformation temperature and strain rate on the tensile properties at high temperature will be investigated. The effects of aging temperature on the creep properties will also be studied.

## 2. Materials and Experiments

**Figure 1:**
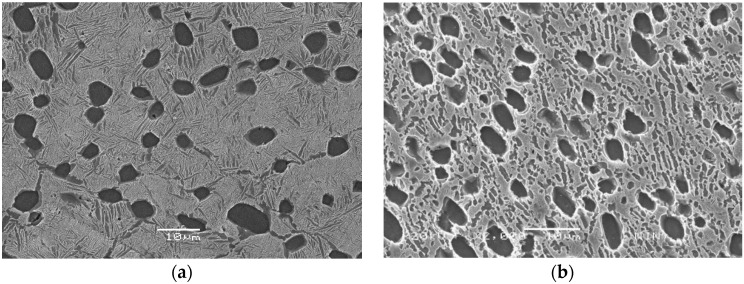
(**a**) Microstructure of the as-forged alloy bar; (**b**) microstructures of the isothermal forged bar in the α_2_ + O+ B2 phase region (the black equiaxed particles are α2 phases, the gray regions are O phases, and light regions are B2 phases).

As-received Ti-22Al-25Nb alloy bar, with dimension Φ240 mm × 360 mm, was provided by the Central Iron and Steel Research Institute (CISRI). The B2 phase transus temperature was measured as 1060 °C by differential thermal analysis (DTA) [7]. This result was consistent with the transus in the Ti-22Al-xNb phase diagram, shown by Boehlert et al. [16]. Chemical analysis showed that the composition of the alloy was consistent with the nominal composition (Table 1), and had lower gas impurity (e.g., oxygen ≤ 500 ppm, hydrogen ≤ 300 ppm, and nitrogen ≤ 800 ppm). The true chemical composition of the alloy was measured with a chemical analysis method, which was Ti-10.8Al-43.0Nb-0.050O-0.007C-0.005N-0.004H in weight percent (wt.%). From Figure 1a, it can be seen that the microstructure of the bar was composed of equiaxed particles, lamellar O phase, and B2 matrix. The equiaxed particles contained α_2_ and rim-O phases. The rim-O phase was formed by the peritectoid reaction of α_2_ phase and B2 phase matrix [7]. The lamellar O phase was formed during air cooling after isothermal forging. The grain size of the B2 phase in the bar was measured to be 38 ± 3 μm. To obtain homogeneous equiaxed particles, the bar was isothermally forged at α_2_ + B2 + O phase regions (980 °C) (Figure 1b). This microstructure contained the equiaxed α_2_ phase, rim-O phase, and lamellar and secondary acicular O phases. Compared with the microstructure of the as-forged bar, more homogeneous equiaxed particles were presented in the B2 matrix.

To study the tensile and creep behaviors of the alloys, the samples were solution-treated at 960 °C for 1 h by water quenching (WQ) and age-treated at 760 °C (HT-760), 800 °C (HT-800), or 840 °C (HT-840) for 12 h followed by air cooling (AC). To observe and analyze the microstructures, standard mechanical polishing methods were used. The heat-treated samples were mechanically polished using a standard metallographic method. Specimens were polished metallographically and etched chemically using HF:HNO_3_:H_2_O (1:3:5). The microstructures of the alloys were characterized by scanning electron microscope (SEM) (Hitachi SU 3500, Tokyo, Japan). The grain sizes of different B2 phases were determined by image analysis on scanning electron micrographs using Image-Pro Plus software (version 6.0) (Image-Pro Plus is a trademark of Media Cybernetics, Inc., Rockville, MD, USA) on optical microscopy and SEM images. Approximately ten images were selected for each test condition. The morphology of O phase was described as the parameter Feret Ratio (Feretmax/Feretmin) (FT). The equiaxed particle and lath can be distinguished by the FT [14]. Due to random orientation distribution of the O lath in three-dimensional spaces, it was not possible to distinguish between width and thickness in the metallographic cross sections. In addition, the actual thickness of the O lath could not be determined because of the angle of the lath inclination to the metallographic surface. The reported mean width values in the work are statistical values of thickness and width of the lamellae in a two-dimensional section of the microstructure.

More details of the microstructure after tensile and creep testing were characterized using TEM with a model JEM-200CX at 200 kV (JEOL, Tokyo, Japan). The TEM samples were prepared through the following steps. First, the samples were cut from the alloys after heat treatment state and creep deformation. Then, they were thinned down to 30–50 μm by polishing with SiC paper. Finally, they were punched to 3-mm disk samples. The electrochemical polishing is the final stage of thinning in a solution of 6% perchloric acid, 34% *n*-butanol and 60% carbinol at −30 °C, with an applied current of 70 mA and a voltage of 30 V.

The diameter of the room temperature tensile samples was 12 mm, and the gauge length was 70 mm. This experiment was conducted using an Instron 1185 mechanical testing machine (Boston, MA, USA). The specimens were deformed at a constant nominal strain rate of 2 × 10^−4^ s^−1^. In order to further evaluate the tensile properties of alloys, the square high temperature tensile specimens were used. The high temperature tensile specimens were 2 mm in thickness, 3 mm in wide and 10 mm in gauge length. The deformation behavior of the alloy was investigated using uniaxial tensile tests on an Instron 5500R machine (Boston, MA, USA), which is attached with a high temperature furnace. The initial dimensions of the high temperature tensile specimens were 1.5 mm in thickness, 3 mm in width, and 10 mm in gauge length. To minimize oxidation, the gauge section of each specimen was coated with glass slurry. To preserve the microstructure under testing temperature conditions, the specimens were rapidly water quenched. These samples were cut from the fracture surface perpendicular to the stretched direction. Before SEM analysis, the fracture surfaces were cleaned with the acetone, ethanol and then water. SEM samples were cut from the undeformed area of tensile samples along the stretched direction. SEM samples were cut from the deformed area of tensile samples perpendicular to the stretched direction. Before SEM analysis, all the SEM samples were electrolytic polished with a solution of 6% perchloric acid, 34% *n*-butanol and 60% carbinol at −30 °C, with an applied current of 70 mA and a voltage of 30 V. The calibrated extensometer was employed in the tensile testing. Three specimens were examined for each combination of solution heat treatment and aging temperatures. Tensile deformations at high temperature were conducted at the temperature of 600–700 °C, and strain rate of 0.001–0.1 s^−1^. The RD creep testing machine (Changchun Kexin Test Instrument Co., Ltd., Changchun, China) was used in this study. The specimens for RD creep tests were prepared by machining 12-mm diameter rods from the heat treated rectangular bars. The RD creep tests were carried out at the temperatures of 650 °C, and the load of 100 N. The specimen was clamped between the upper and lower dies, and was loaded through a Si_3_N_4_ spherical ball indenter of diameter 2.38 mm. In order to prevent severe oxidation of the specimen, the tests were carried out in high purity argon gas atmosphere. The specimen temperature was maintained constant with an accuracy of ±1 °C.

## 3. Results and Discussion

### 3.1. Microstructures

**Figure 2:**
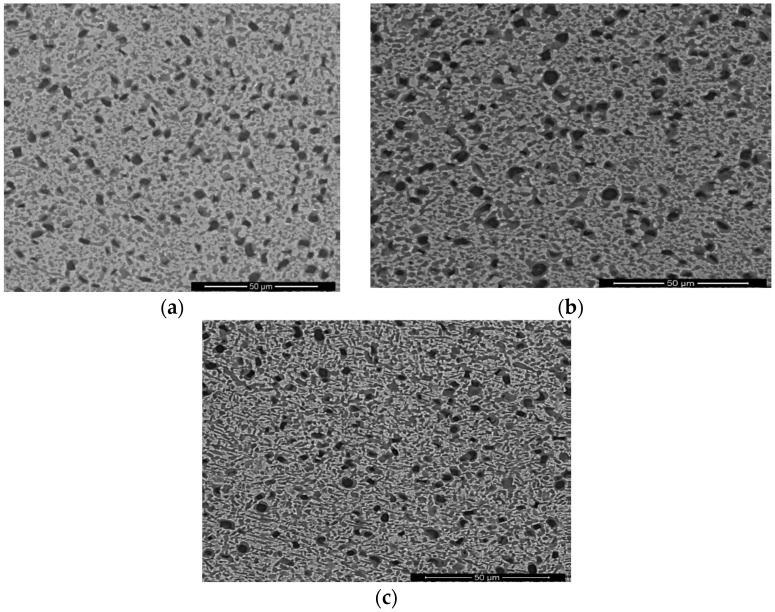
Comparison of a 960 °C/1 h solution-treated specimen and subsequently were aged at different temperatures. The specimens were aged at (**a**) 760 °C, (**b**) 800 °C, (**c**) 840 °C.

Figure 2a–c is the microstructures of three different heat-treated samples. All microstructures contained α_2_ phase particles, lamellar O phase, secondary acicular O phase, and B2 matrix. It can be seen that they are very similar, except for the differences in the secondary acicular O phase. All the aged microstructures contained secondary acicular O phase within the B2 matrix. That is because the O and B2 phases have specific orientation relationships (O.R.) in the low-energy configuration [12]. The statistical data of microstructural characteristics in the three microstructures are listed in Table 2. In Table 2, the standard deviations have provided with the mean values. It can be seen that as aging temperature increased, the diameter of equiaxed α_2_ phase showed nearly unvaried value, the volume fractions of total precipitates and secondary acicular O phase, and width of the secondary acicular O phase increased, while the length of acicular O decreased. Hence, the secondary acicular O phases were determined by aging temperature. The thickest size of secondary acicular O phase was presented at 840 °C, and the thinnest size was displayed at 760 °C. From our previous results in our group [14], the equiaxed particles in these microstructures were determined by isothermal forging temperature. The lamellar O phases were decided by solution temperature, and the secondary acicular O phases were adjusted by aging temperature. As aging temperature increased, the thickness of secondary acicular O phase increased, while the length and volume fraction decreased. The major research objectives in this work were tensile and creep behaviors, and the deformation mechanisms of the three microstructures in the latter.

### 3.2. Room Temperature Tensile Properties and Deformation Mechanism

Tensile properties of the equiaxed microstructure at ambient temperature are presented in Table 3. In Table 3, the standard deviations have provided for these mean values of the four mechanical properties. In most cases the results represent the average of at least three tests. Thus, the tensile strength of HT-760 was better than that of HT-840. However, it exhibited worse elongation and reduction area.

Strengthening mechanisms of Ti_2_AlNb-based alloys are similar to those of traditional titanium alloys. The various parameters that influence the yield strength are: (1) grain size of the B2 phase, (2) strengths of the individual phases influenced by changes in their composition with heat-treatment, (3) volume fractions of α_2_/O and B2 phases, (4) lath size of O phase, and (5) dislocation substructure [17]. It can be seen that grain boundary strengthening is the most important factor. The sizes of the secondary acicular O phase should be another important factor. In the three microstructures, aging temperatures are varied. Previous results showed that equiaxed particles were regulated by isothermal forging, lamellar O phases were controlled by varied solution temperature, and the secondary acicular O phases were adjusted by the aging temperature. The coarsening of secondary acicular O phases was caused by increasing aging temperature. Thus, the tensile mechanical properties of three microstructures were mainly decided by the secondary acicular O phases. This variation trend of strength is caused by noticeable coarsening and volume fraction reduction of secondary acicular O phase with increasing aging temperature [18]. This result is in agreement with literature [15]: that the yield strength is associated with the sizes and morphologies of constituent phases.

Equiaxed α_2_ phase, B2 phase, and the region around the α_2_ and O phases are the major factors influencing the variation in ductility. The differences in *E* value are mainly ascribed to the volume fractions of the O phase and B2 phase. The O-phase has fewer available slip systems than the B2 structure [16]. During tensile deformation, the ductility of the alloy was determined by the number of slip systems. Compared with B2 phase, O phase has fewer slip systems, thus the lower *E* value was attributed to the limited slip systems in O-phase-dominated microstructures. The microstructure of HT-840 has more B2 phases and less acicular O phases, so the ductility of HT-840 at room temperature was superior to that of HT-760.

**Figure 3:**
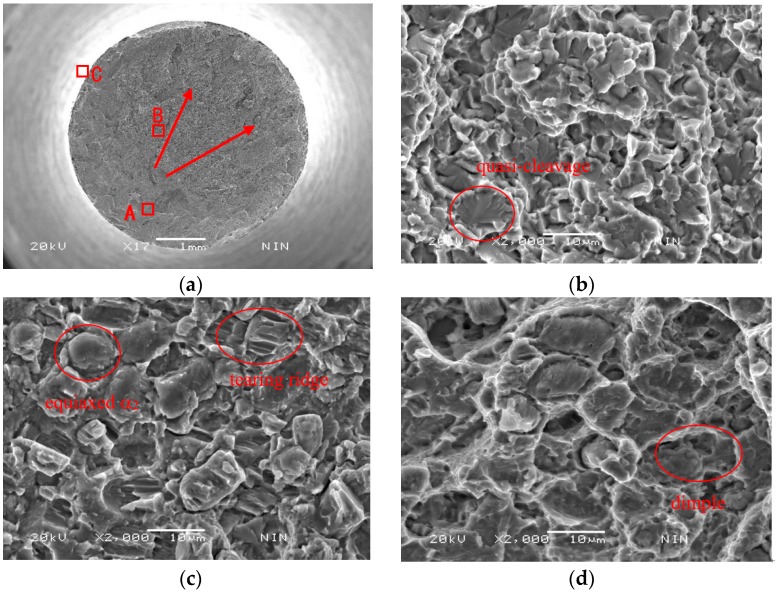
Room-temperature tensile fractographs of equiaxed-microstructure (the tensile samples were cut from the tangential direction of the circular billets which were formed through isothermally forging at α_2_ + B2 + O phase regions), (**a**) macro-fractograph (area A is the fibrous zone, area B is the radiation area and area C is the shear lip); (**b**) the enlarged micro-fractograph of the red box in area A; (**c**) the enlarged micro-fractograph of the red box in area B; (**d**) the enlarged micro-fractograph of the red box in area C.

In the following discussion, room temperature (RT) tensile deformation mechanism of equiaxial microstructure was further analyzed by SEM and TEM techniques. Figure 3 is the tensile fractographs of HT-760. The fractographs contain the fibrous zone (A in Figure 3a), radiation area (B in Figure 3a), and the shear lip (C in Figure 3a). The shear lip of the fracture face was smallest. From Figure 3, it can be seen that a crack started from the specimen’s interior (fibrous zone). The orientation of the crack growth was presented along the arrows of Figure 3a. As the stress increased to critical value, the crack was propagated rapidly. The shear lip was formed on the rim of the fracture. Figure 3c is the microstructure of the radiation area. The size of the raised particle is similar to that of the equiaxed α_2_ phase. The tear ridge of the equiaxed α_2_ particles showed the shear fracture characteristics. The explanation for this is that the deformed abilities of different phases were varied during tensile tests. The slip systems in equiaxed α_2_ phase was less than that of B2 matrix, thus B2 matrix has better deformability. Due to different deformability of each phases, the microvoids were easily formed at phase interface between equiaxed α_2_ phase and B2 matrix. The shear dimples were formed along the direction of shearing stress (Figure 3d). From above analysis, the room temperature fracture mechanism of HT-760 is a mixed-rupture characteristic of quasi-cleavage and dimples.

**Figure 4:**
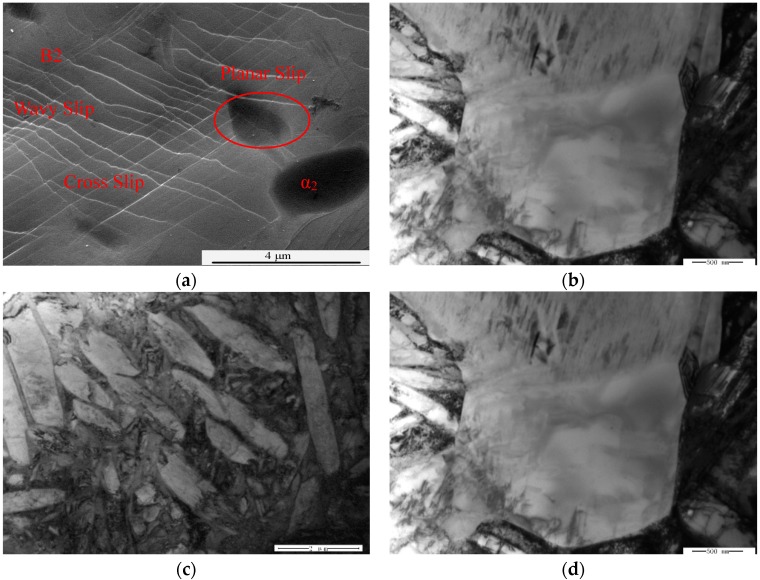
(**a**) Surface slip lines of the deformation area in tensile sample; (**b**) Equiaxed particles in undeformed zone of tensile sample; (**c**) the dislocation morphology of lamellar and equaxied α_2_ particles in deformed zone of tensile sample; (**d**) slip band in equaxied α_2_ particles in deformed zone of tensile sample.

The surface slip characteristics of the deformed samples could reflect the deformation mechanism of samples. Figure 4a is the micrograph of surface slips in HT-760. Wavy slip lines were present in the B2 phase. Meanwhile, longer slip lines were passed through equiaxed α_2_ particles and B2 matrix. This phenomenon showed that cross-slips and multiple slips operated in the B2 phase. There are longer slip lines in the equiaxed α_2_ phase—this means that plane slippage characteristics were present in the α_2_ phase. B2 phase has more slip planes than the α_2_/O phase, thus, deformation was mainly focused in the B2 matrix [19,20]. To further explore the RT deformation mechanism of HT-760, the microstructures were studied by TEM. In the undeformed area of tensile samples (Figure 4b), the dislocation density in the interior of the equiaxed α_2_ phase was lower, while the dislocation density at phase interfaces between B2 and O was higher. The explanation for this is that during isothermal forging, the large deformation was occurred at phase interfaces between α_2_/O and B2 phases. After tensile deformation (Figure 4c), dislocation of the primary α_2_ phase was straight, and formed a dislocation net. Dislocation pile-up and tangling around the lamellar O were also observed after a high degree of deformation. Multi-slips were activated in the equiaxed α_2_ particles. In the region of large plastic strain, the dislocation net formed by dislocation slips was passed through all equiaxed particles. It could relieve high stress in the particles, and coordinate deformation between the equiaxed α_2_ particles and B2 matrix (Figure 4d).

**Figure 5:**
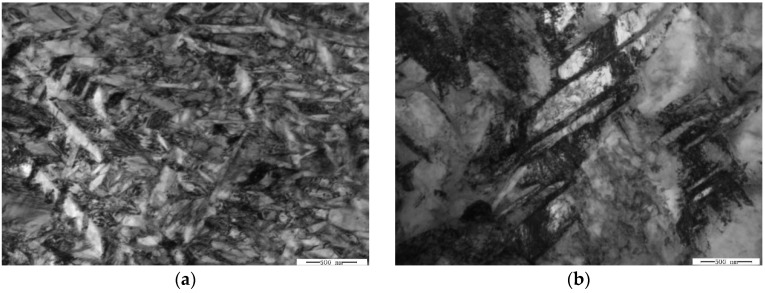
Lamellar O phase in tensile sample (the samples were cut from the tangential direction of the circular billets which were formed through isothermally forging at α_2_ + B2 + O phase regions. The TEM samples were cut from the tensile sample at the deformation area along the stretched direction), (**a**) the lamellar O phase in the undeformed zone of tensile sample; (**b**) slip band of the lamellar O phase in the deformation zone of tensile sample.

In the equiaxed microstructure, besides the equiaxed α_2_ particles, the influence of lath O phases on RT tensile properties is also important. Figure 5 is the undeformed and deformed lath O phases. The undeformed lamellar O phase has lower dislocation density (Figure 5a). Figure 5b is the deformed lamellar O phase, it can be seen that slip bands originated at the interface between B2 and O phases, and then passed through the lamellar O phase and B2 matrix. There were many piled up dislocations at the phase interface between O and B2. Slip bands such as B2 → O → B2 were also observed, because the slip bands from the B2 phase were went through the lamellar O phase and B2 matrix. The continuous B2 phase promoted the development of slip systems. This is the key factor for improving the ductility of this alloy (Figure 5b). Based on the above analysis about the deformation of the equiaxed microstructure, the mechanisms of nucleation and propagation of cracks could be concluded. In the equiaxed microstructure, slip bands were transferred via equiaxed particles, cracks were easily formed at the interface between the equiaxed α_2_ phase and B2 phase.

### 3.3. High Temperature Tensile Deformation

**Figure 6:**
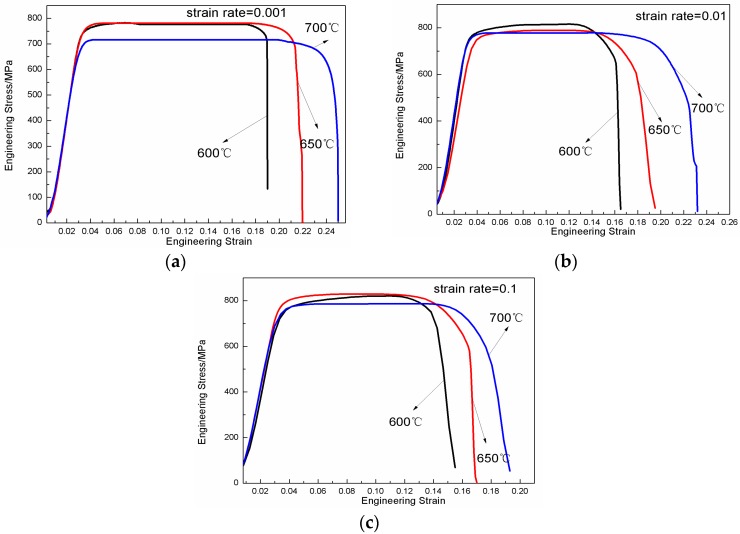
Engineering stress-engineering strain curves of the tensile samples at different temperatures and strain rates. (The initial dimensions of the high temperature tensile specimens were 1.5 mm in thickness, 3 mm in width and 10 mm in a gauge length. To minimize oxidation, the gauge section of each specimen was coated with glass slurry. To preserve the microstructure under testing temperature conditions, the specimens were rapidly water quenched.), (**a**) 0.001 s^−1^, (**b**) 0.01 s^−1^, (**c**) 0.1 s^−1^.

Figure 6 shows the engineering stress-strain curves of HT-760 at different temperatures and strain rates. The curves present two characteristics. Firstly, the deformation temperature is sensitive to the flow stress. As the temperature increased, the strength of alloy decreased, while the ductility increased. At the strain rate of 0.001 s^−1^, the deformation temperature increased from 600 to 700 °C, as the peak stress decreased from 778 to 712 MPa. Meanwhile, the flow stresses of the alloys also decreased. Secondly, at constant strain rate, the ductility of the alloy improved with increasing deformation temperature. The main reason is that (a) before tensile deformation, the samples needed to be heated to the deformation temperature. The temperature range of 600–700 °C belongs to the O + B2 phase region. As more secondary acicular O phases were precipitated from the B2 matrix, the relative content of B2 phase decreased, which is a process of precipitation strengthening. Another reason is that (b) the activation energy of the alloy also increased with the increasing deformation temperature. Hence, the flow stress of the alloy decreased while the ductility increased.

**Figure 7:**
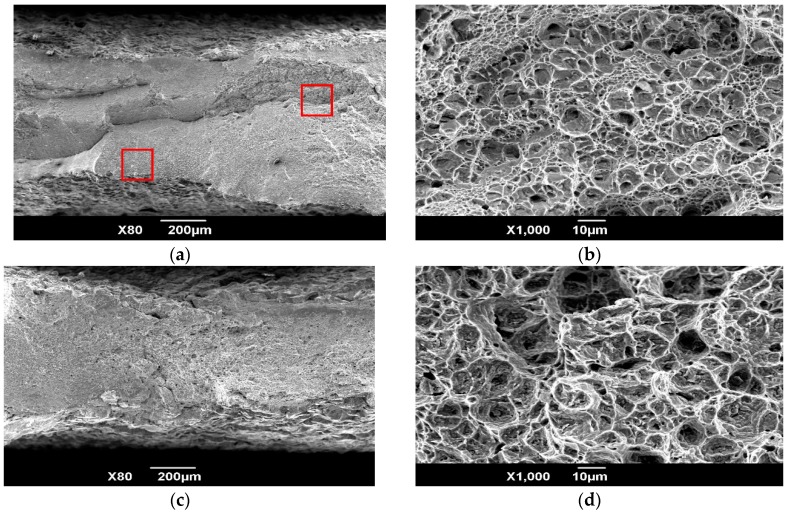
High temperature fractograph under the different deformation temperatures with strain rates of 0.01 s^−1^, (**a**) macro-fractograph at the cross section of the 600 °C tensile samples; (**b**) the enlarged micro-fractograph of the red box in Figure 7a; (**c**) macro-fractograph at the cross section of the 700 °C tensile samples; (**d**) the enlarged micro-fractograph of the red box in Figure 7c.

**Figure 8:**
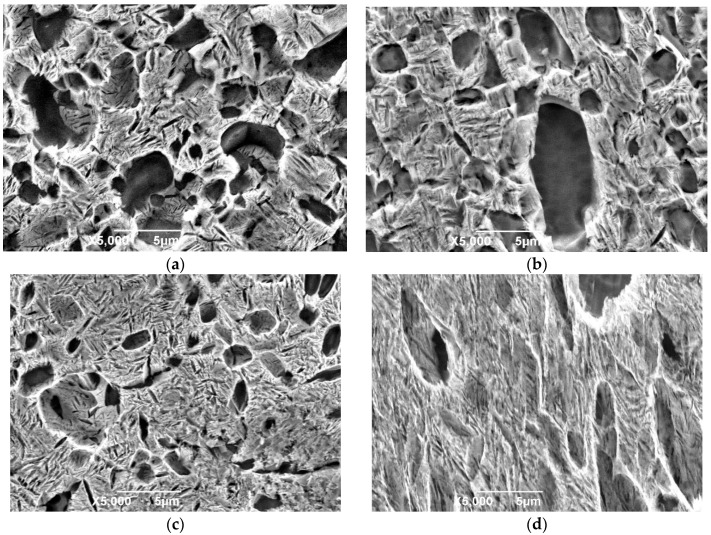
Microstructures of the deformed and undeformed parts in tensile samples under the different strain rates with deformation temperatures of 700 °C, (**a**) microstructure of the undeformed parts at the strain rate of 0.001 s^−1^; (**b**) microstructure of the deformed parts at the strain rate of 0.001 s^−1^; (**c**) microstructure of the undeformed parts at the strain rate of 0.1 s^−1^; (**d**) the microstructure of the deformed parts at the strain rate of 0.1 s^−1^.

Figure 7 shows high-temperature fractographs under the different deformation temperatures, with strain rates of 0.01 s^−1^. The fracture appearance shows that ductile fracture occurred during high temperature deformation. From the microscopic morphology, it can be seen that the dimple pattern gradually increased with increasing test temperature, meaning that the ductility of the alloy increased. The microstructure evolution under the different strain rates with deformation temperatures of 700 °C are presented in Figure 8. Relative to the heat-treated microstructure, the undeformed zones were heat-treated again and had more acicular O phases. In the deformed area, as the strain rate increased, the volume fraction of O phases was nearly constant, owing to the attainment of phase equilibrium, while the lamellar O phase was not stable and inclined to the tensile direction. Therefore, the microstructural evolution during deformation was dominated by phase and morphology changes.

### 3.4. Creep Properties

**Figure 9:**
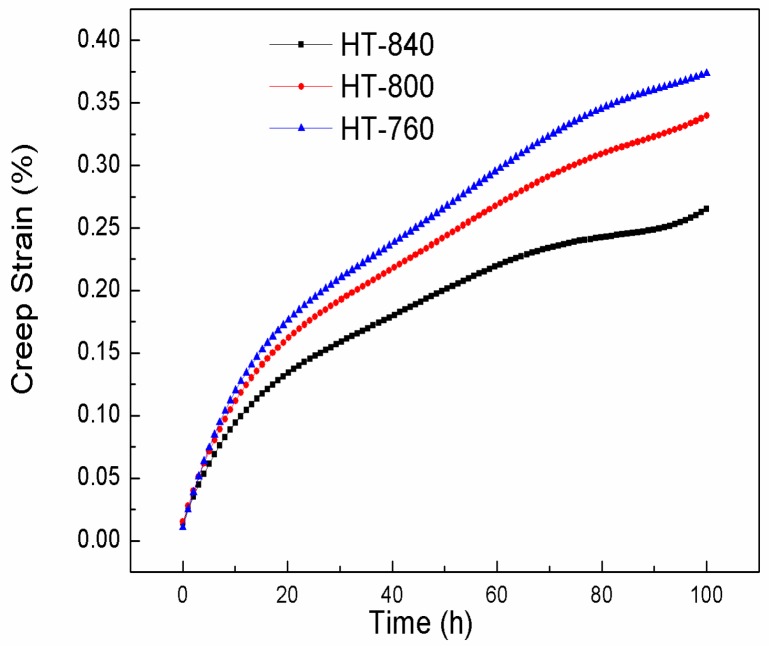
Creep curves at the stress of 150 MPa and temperature of 650 °C.

The creep properties of three microstructures were also investigated. Creep curves at the stress of 150 MPa and temperature of 650 °C are showed in Figure 9. As the aging temperature increased, the creep resistances of the microstructures also improved. The creep rate of the primary creep stage for HT-840 was lower than those of HT-800 and HT-760. HT-840 had the best creep resistance, at 650 °C/150 MPa. The reasons for different creep properties in three heat treatment schedules are the differentiation of microstructure before creep deformation. The microstructure after isothermal forging mainly contained equiaxed α_2_ particles and the lamellar O phase formed by air cooling. After solution treatment, the acicular O phase was dissolved in B2 matrix. Some had already become globular during heat-treatment. During aging treatment processing of the O + B2 phase region (760–840 °C), very fine secondary acicular O phases were presented at 760 °C. This acicular O phase easily deformed during creep deformation; thus, the creep resistance of HT-760 was worst. The coarse lamellar phase formed at high aging treatment temperature (840 °C) had the function of strengthening during creep deformation.

**Figure 10:**
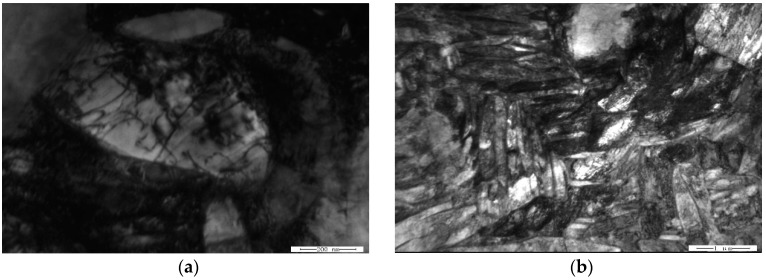
TEM images of creep sample HT-760 at 650 °C/150 MPa, (**a**) α_2_ particles, (**b**) lamellar O phase.

**Figure 11:**
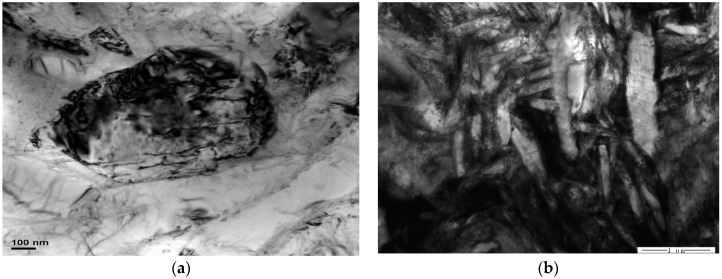
TEM images of creep sample HT-840 at 650 °C/150 MPa, (**a**) α_2_ particles, (**b**) lamellar O phase.

Figure 10 and Figure 11 are the microstructures of HT-760 and HT-840, respectively, after creep deformation. When the aging temperature increased, the dislocation density of the equiaxed α_2_ particles reduced. In the HT-760 microstructure, there were several dislocation pile-ups at the interfaces of equiaxed α_2_ particles and lamellar O phase. Some dislocations passed through the interiors of equiaxed particles, reducing the creep resistances of the equiaxed particles. For the HT-840 microstructure, dislocation hardly went around the coarser lamellar O phase, while the secondary acicular O phase was smaller and more easily deformed. Boehlert and Miracle [21] overviewed the creep behaviors reported for α_2_- and O-based Ti-Al-Nb alloys. They divided the creep behaviors into three regimes, and identified the creep mechanism in each regime according to the values of *n* and *Q*. When *n* = 1 to 1.9 and *Q* = 107 to 187 kJ/mol, the creep process is controlled by grain-boundary diffusion. When *n* = 1.9 to 2.8 and *Q* = 256 to 327 kJ/mol, the creep process is controlled by grain-boundary sliding. When *n* = 3.5 to 7.2 and *Q* = 241 to 376 kJ/mol, the creep process is controlled by dislocation climb. It is generally accepted that high-temperature dislocation climb is associated with lattice self-diffusion, while grain-boundary sliding is associated with either lattice self-diffusion or grain-boundary diffusion. Typically, the activation energy for grain-boundary diffusion is half that of lattice self-diffusion [22,23,24]. It is no denying that diffusion has an important influence on creep properties of alloys. During current research, the creep mechanism of equiaxed microstructure is therefore dominated by a dislocation-controlled creep process. This conclusion is supported by observations of dislocation densities. The current observations are insufficient to determine whether a glide or climb mechanism is rate controlling.

## 4. Conclusions

This study investigated tensile deformation mechanism, and creep behavior of the equiaxed microstructure. The equiaxed microstructures were formed by isothermal forging in the α_2_ + B2 + O phase region, and then heat treatment in the α_2_ + B2 + O and B2 + O phase regions. The rim-O can be obtained via peritectoid reaction of B2 and α_2_ phases and it can improve glide deformation of the particle to enhance hot workability. The secondary acicular O phase can be controlled by aging treatment. Coarsening of the lamellar O phase was caused by increasing aging temperature.

The yield strength and ultimate tensile strength of HT-760 outperformed HT-840 at RT, however the *E* and RA of HT-760 were lower. Deformation of the alloys mainly depended on the volume fraction and deformation of the B2 phase. During high temperature deformation at constant strain rate, the flow stress decreased, and the ductility increased as the deformation temperature increased. At the same deformation temperature, as the strain rate increased the strength increased and the ductility decreased.

The creep rate of HT-840 was lower than those of HT-800 and HT-760. HT-840 had the best creep resistance at 650 °C/150 MPa. This is because this acicular O phase was easily deformed during creep deformation, while the coarser lamellar O phase at the higher aging treatment temperature (840 °C) had the function of strengthening during creep deformation.

## Figures and Tables

**Table 1:** Chemical composition of the Ti-22Al-25Nb alloy (at.%) (The true chemical composition of the alloy was measured with a chemical analysis method, which was Ti–10.8Al–43.0Nb–0.050O–0.007C–0.005N–0.004H in weight percent (wt.%)).

Ti	Al	Nb	O	N	H
Bal	22.3	25.7	0.00043	0.000052	0.000009

**Table 2:** The main parameters of the microstructures during various heating treatment. *V*_total_ (%), *V*_acicular_ (%), *L*_acicular_ (μm), *W*_acicular_ (μm) and *d*_equiaxed_ (μm) are the volume fraction of total precipitates, acicular O phase, the length of acicular O, the width of acicular O, and the diameter of equiaxed particles, respectively. ε is the average errors.

S.T./°C	A.T./°C	*V*_total_ (±ε)	*V*_acicular_ (±ε)	*L*_acicular_ (±ε)	*W*_acicular_ (±ε)	*d*_equiaxed_ (±ε)
	760	38.63 ± 1.8	1.64 ± 0.25	2.83 ± 0.26	0.13 ± 0.003	4.27 ± 0.16
960	800	43.71 ± 2.1	2.58 ± 0.32	2.19 ± 0.21	0.31 ± 0.007	4.62 ± 0.18
	840	50.73 ± 2.3	3.94 ± 0.37	1.64 ± 0.18	0.45 ± 0.009	5.03 ± 0.22

S.T. = solution treatment; A.T. = age treatment.

**Table 3:** Room temperature tensile properties of the Ti-22Al-25Nb alloy (at.%).

Sample	S.T./°C	A.T./°C	Ultimate Tensile Strength/MPa	Yield Strength/MPa	Elongation/%	Reduction Area/%
HT-760	940	760	1097	1004	9.5	12.5
HT-800	940	800	1060	998	11.5	15.5
HT-840	940	840	1047	966	12	15.5

S.T. = solution treatment; A.T. = age treatment.

## References

[1] Banerjee D., Gogia A.K., Nandi T.K., Joshi V.A. (1988). A new ordered orthorhombic phase in a Ti_3_Al-Nb alloy. Acta Metall..

[2] Cowen C.J., Boehlert C.J. (2006). Microstructure, creep, and tensile behavior of a Ti-21Al-29Nb (at.%) orthorhombic + B2 alloy. Intermetallics.

[3] Lin P., He Z., Yuan S., Shen J., Huang Y., Liang X. (2013). Instability of the O-Phase in Ti-22Al-25Nb alloy during elevated-temperature deformation. J. Alloy. Compd..

[4] Xue C., Zeng W., Xu B., Liang X., Zhang J., Li S. (2012). B2 grain growth and particle pinning effect of Ti-22Al-25Nb orthorhombic intermetallic alloy during heating process. Intermetallics.

[5] Sun Y., Zeng W., Ma X., Xu B., Liang X., Zhang J. (2011). A hybrid approach for processing parameters optimization of Ti-22Al-25Nb alloy during hot deformation using artificial neural network and genetic algorithm. Intermetallics.

[6] Wang W., Zeng W., Li D., Zhu B., Zheng Y., Liang X. (2016). Microstructural evolution and tensile behavior of Ti_2_AlNb alloys based α_2_-phase decomposition. Mater. Sci. Eng. A.

[7] Wang W., Zeng W., Xue C., Liang X., Zhang J. (2014). Microstructural evolution, creep, and tensile behavior of a Ti-22Al-25Nb (at.%) orthorhombic alloy. Mater. Sci. Eng. A.

[8] Peng J., Li S., Mao Y., Sun X. (2001). Phase transformation and microstructures in Ti-Al-Nb-Ta system. Mater. Lett..

[9] Dang W., Li J., Zhang T., Kou H. (2015). Microstructure and phase transformation in Ti-22Al-(27-*x*) Nb-*x*Zr alloys during continuous heating. J. Mater. Eng. Perform..

[10] Małecka J. (2015). Investigation of the oxidation behavior of orthorhombic Ti_2_AlNb alloy. J. Mater. Eng. Perform..

[11] Jia J., Zhang K., Lu Z. (2014). Dynamic recrystallization kinetics of a powder metallurgy Ti-22Al-25Nb alloy during hot compression. Mater. Sci. Eng. A.

[12] Peng J., Mao Y., Li S., Sun X. (2001). Microstructure controlling by heat treatment and complex processing for Ti_2_AlNb based alloys. Mater. Sci. Eng. A.

[13] Cowen C.J., Boehlert C.J. (2007). Comparison of the microstructure, tensile, and creep behavior for Ti-22Al-26Nb (At. Pct) and Ti-22Al-26Nb-5B (At. Pct). Metall. Mater. Trans. A.

[14] Wang W., Zeng W., Xue C., Liang X., Zhang J. (2014). Quantitative analysis of the effect of heat treatment on microstructural evolution and microhardness of an isothermally forged Ti-22Al-25Nb (at.%) orthorhombic alloy. Intermetallics..

[15] Wang W., Zeng W., Xue C., Liang X., Zhang J. (2015). Microstructure control and mechanical properties from isothermal forging and heat treatment of Ti-22Al-25Nb (at.%) orthorhombic alloy. Intermetallics.

[16] Boehlert C.J., Majumdar B.S., Seetharaman V., Miracle D.B. (1999). Part I. The Microstructural evolution in Ti-Al-Nb O + Bcc orthorhombic alloys. Metall. Mater. Trans. A.

[17] Emura S., Araoka A., Hagiwara M. (2003). B2 grain size refinement and its effect on room temperature tensile properties of a Ti-22Al-27Nb orthorhombic intermetallic alloy. Scr. Mater..

[18] Wu Y., Hwang S.K. (2001). The Effect of aging on microstructure of the O-phase in Ti-24Al-14Nb-3V-0.5 Mo alloy. Mater. Lett..

[19] Lin P., He Z., Yuan S., Shen J. (2012). Tensile deformation behavior of Ti–22Al–25Nb alloy at elevated temperatures. Mater. Sci. Eng. A.

[20] Hagiwara M., Emura S., Araoka A., Kong B.O., Tang F. (2003). Enhanced mechanical properties of orthorhombic Ti_2_AlNb-based intermetallic alloy. Met. Mater. Int..

[21] Boehlert C.J., Miracle D.B. (1999). Part II. The creep behavior of Ti-Al-Nb O + Bcc orthorhombic alloys. Metall. Mater. Trans. A.

[22] Mishra R.S., Banerjee D. (1990). Microstructure and steady state creep in Ti-24Al-11Nb. Mater. Sci. Eng. A.

[23] Mishra R.S., Banerjee D., Mukherjee A.K. (1995). Primary creep in a Ti-25Al-11Nb alloy. Mater. Sci. Eng. A.

[24] Zhang J.W., Lee C.S., Lai J.K.L., Zou D.X., Li S.Q. (1998). Microstructure and creep behavior of an orthorhombic Ti-25Al-17Nb-1Mo alloy. Metall. Mater. Trans. A.

